# Erosive potential of energy drinks on the dentine surface

**DOI:** 10.1186/1756-0500-6-67

**Published:** 2013-02-19

**Authors:** Shelon CS Pinto, Matheus C Bandeca, Carolina N Silva, Rodrigo Cavassim, Alvaro H Borges, José E C Sampaio

**Affiliations:** 1 Department of Dentistry, Araraquara Dental School, University of Sao Paulo State, Araraquara, Brazil; 2 Ceuma University - UniCEUMA, Sao Luis, Brazil; 3 University of Cuiaba - UNIC, Cuiaba, Brazil

**Keywords:** Dentine hypersensitivity, Erosive tooth wear, Scanning electron microscopy, Dentinal tubules, Abrasion, Tooth wear, Root dentin, Toothbrushing

## Abstract

**Background:**

Considering the current high consumption of energy drinks, the aim of the present study is to evaluate the influence of energy drinks in removing the smear layer and exposing dentinal tubules on root surface.

**Methods:**

Dentine root surfaces were exposed using a diamond bur. Forty movements of scaling were performed in the area prepared in order to create a smear layer. One hundred and thirty specimens were obtained from 35 teeth. Specimens were randomly distributed into 12 groups (n = 10) and divided into subgroups according to the application: topical (n = 5) and friction (n = 5). Twelve energy drinks were evaluated: RedBull™, Burn™, TNT™, Flash Power™, Flying Horse™, Sports Drink™, Ionic™, Hot Power™, Army Power™, Gladiator™ and Bug™. Distilled water was used as a control group. The specimens were analysed by scanning electron microscopy.

**Results:**

Topical application: a significant influence of energy drinks on smear layer removal was found for FlyingHorse™ and Bug™ when compared with the control group. Friction application: significant smear layer removal was found for Burn™, FlyingHorse™, Gladiator™, SportsDrinks™, when compared with the control group. Comparing the different application forms, a statistically significant difference was found for Army Power™.

**Conclusion:**

Considering the significant smear layer removal, energy drinks can be an important etiological factor for cervical dentine hypersensitivity.

## Background

Cervical hypersensitivity is a dental problem that has increased by several factors; one of them is due to the indiscriminate use of soft drinks [1-3]. Soft drink intake is the most important factor related to dentinal tubule exposure followed by dentine hypersensitivity, since the consumption of acidic drinks has increased considerably [4-6]. After root dentine exposition, dentine wear can be easily provoked by erosion and/or abrasion [7-10]. One important extrinsic factor in erosive tooth wear is the high consumption of energy drinks [11].

Energy drinks are basically soft drinks with some forms of vitamins and other chemicals that increase energy for a very short period [10]. These drinks have been developed in order to increase physical resistance and the state of alertness. In addition, they increase concentration, stimulate metabolism and help to eliminate harmful substances from the body [12].

According to Cavalcanti et al. [11], energy drinks have a high erosive potential, as they have low pH and a high non-reducing sugar content. Nevertheless, this *in vitro* study assessed the physical-chemical characteristics of them; their influence on the tooth surface has not been evaluated. Therefore, the aim of the present *in vitro* study was to evaluate the influence of energy drinks in removing smear layer and subsequent dentinal tubules exposure on root surface. The null hypothesis tested was that the energy drinks do not expose the dentinal tubules on the root surface.

## Methods

This study was approved by the Sao Paulo State University – Araraquara Dental School (UNESP – FOAr) Ethical Committee (06534-9/2010).

### Specimen preparation

Thirty-five third molar human teeth were used in this investigation. Teeth were obtained from the Human Teeth Bank of Sao Paulo State University – Araraquara Dental School.

Two parallel grooves 0.5 mm deep on the vestibular and lingual root surfaces of each tooth were performed: one at the cementoenamel junction and the other 4 mm apical to the CEJ. The area between the two grooves was flattened with the same bur (Diamond bur - 2135). Forty movements of scaling were performed in the area prepared, with a Gracey curette (Gracey Instruments™ ) in order to create a smear layer.

The roots were cross cut in the first groove, in order to remove the crown. Four dentine blocks, approximately 2 × 2 mm, were obtained from each tooth, two from the vestibular and two from the lingual surface. All dentine blocks were observed on stereomicroscope with × 25 magnification to check for the presence of cracks on their surfaces. After analysing the surfaces, twenty dentine blocks with presence of cracks were removed.

### Experimental groups

Specimens were randomly distributed into 12 groups (n = 10). Ten specimens were used in each group. The specimens were divided in subgroups according to the application: topical (n = 5) and friction (n = 5). Eleven energy drinks were evaluated: RedBull™ (RedBull GmbH, Austria), Burn™ (Coca-Cola), TNT™ (Refrix Ltda), Flash Power™ (Alflash Ltda), Flying Horse™ (NewAge Ltda), Sports Drink™ , Ionic™ (Capucci & Barra.com Ltda), Hot Power™ (Ultrapan Ltda), Army Power™ (Capucci & Barra.com Ltda), Gladiator™ (Coca-Cola) and Bug™ (Refrix, Ltda).

Distilled water was used as a control group in order to compare with the dentinal tubules exposure by energy drinks.

#### Subgroups

The groups were divided into 2 subgroups according to the following protocol:

- Topical Application: specimens were immersed for 5 minutes in the beverages, and rinsed with distilled water for 15 seconds.

- Friction Application: specimens were immersed for 5 minutes in the beverages, brushed with an electric toothbrush for 30 seconds (friction), and rinsed with distilled water for 15 seconds.

### pH evaluation at baseline

The pH of each beverage was determined in triplicate at room temperature using a pHmeter (AT-350, Sao Paulo, Brazil).

### Scanning electron microscopy (SEM)

**Figure 1:**
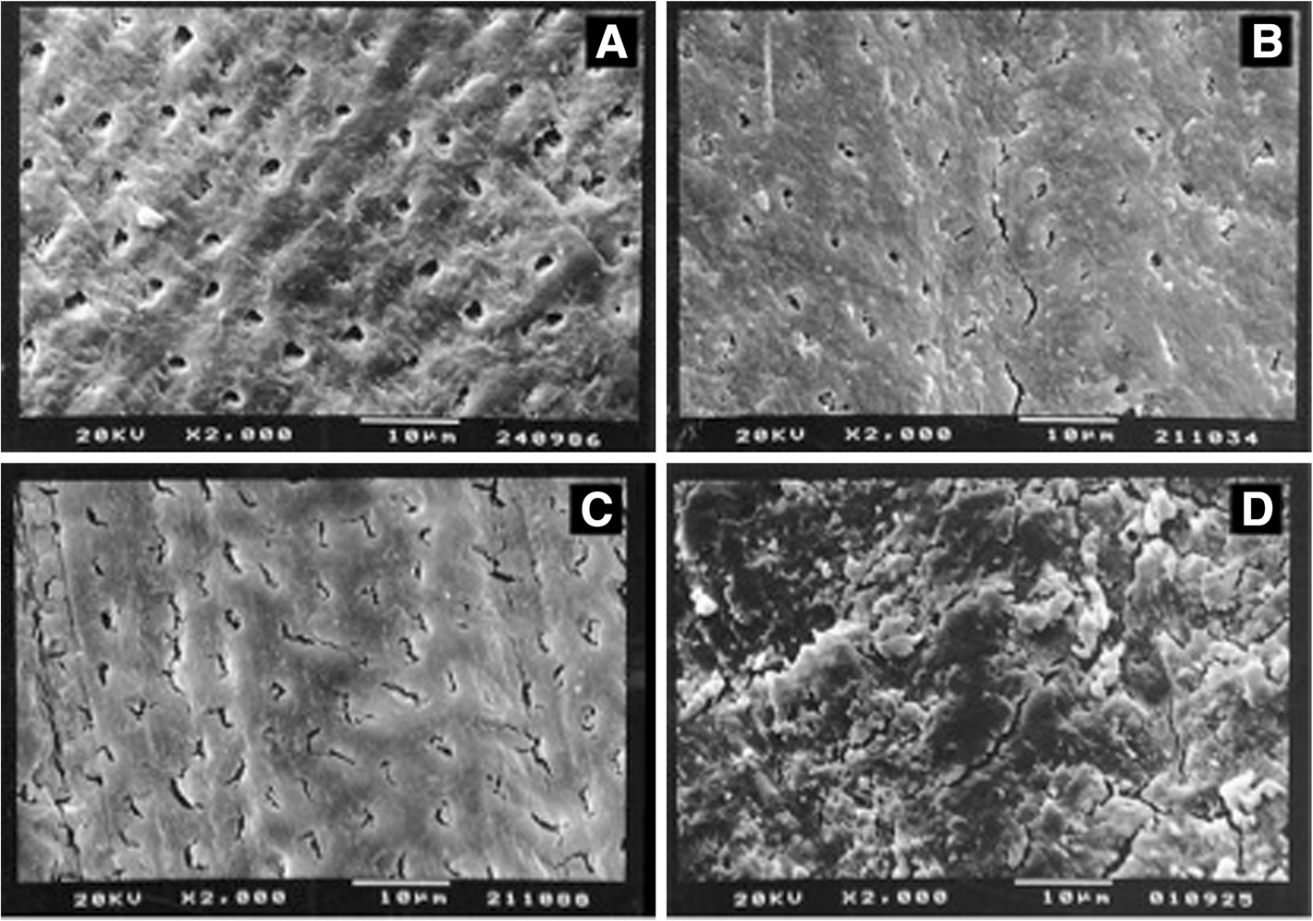
**Scanning electron microscope photomicrographs (magnification of 2,000×) showing the score to verify the dentinal tubules exposure. A**) Grade 1 - Dentinal tubules fully opened; **B**) Grade 2 – Dentinal tubules partially opened; **C**) Grade 3 – Traces of opened dentinal tubules; **D**) Grade 4 – Dentinal tubules totally obliterated.

The magnification of each photomicrograph sample was 500× and 2,000×. All photomicrographs were obtained through a scanning electron microscope (SEM) operated at 20 kV (Jeol JSM). After the photomicrographs were obtained, they were identified and scored as follows to verify the dentinal tubules exposure (Figure 1):

Grade 1 Dentinal tubules fully opened (Figure 1A).

Grade 2 Dentinal tubules partially opened (Figure 1B).

Grade 3 Traces of opened dentinal tubules (Figure 1C).

Grade 4 Dentinal tubules totally obliterated (Figure 1D).

#### Data reproducibility

A single examiner was calibrated by evaluating photomicrographs with predetermined scores in order to accurately measure the four types of dentinal tubules exposure. After calibration, a blinded examiner evaluated the photomicrographs that were evaluated three times at 24-hour intervals. Each sample received the final score that prevailed among the three readings and then intra-examiner reliability was calculated by comparing the three readings and the kappa test for agreement was 0.95.

#### Statistical analysis

The Kruskal-Wallis non-parametric test was used to determine the difference among the groups for the Index of Smear Layer Removal. The Dunn’s multiple comparison test was used to perform pair-wise multiple comparisons. The differences between the application methods were evaluated using the Mann–Whitney test.

## Results

The energy drinks showed pH that was extremely acidic. The lowest level of pH in energy drinks was the Sports Drink (2.52 ± 0.11) and the highest level of pH in energy drinks was the RedBull (3.81 ± 0.21). All pH values of energy drinks are shown in Table 1.

**Table 1:** pH values of the energy drinks

**Group**	**Energy drink**	**pH ± SD**
1	Control (Distilled water)	8.17 ± 0.12
2	RedBull	3.81 ± 0.21
3	Flying Horse	3.35 ± 0.13
4	BUG	2.92 ± 0.11
5	Burn	3.03 ± 0.13
6	Gladiator	2.88 ± 0.16
7	Hot Power	2.87 ± 0.17
8	Flash Power	2.79 ± 0.22
9	Army Power	2.69 ± 0.18
10	Ionic	2.85 ± 0.31
11	TNT	2.80 ± 0.24
12	Sports Drinks	2.52 ± 0.11

**Figure 2:**
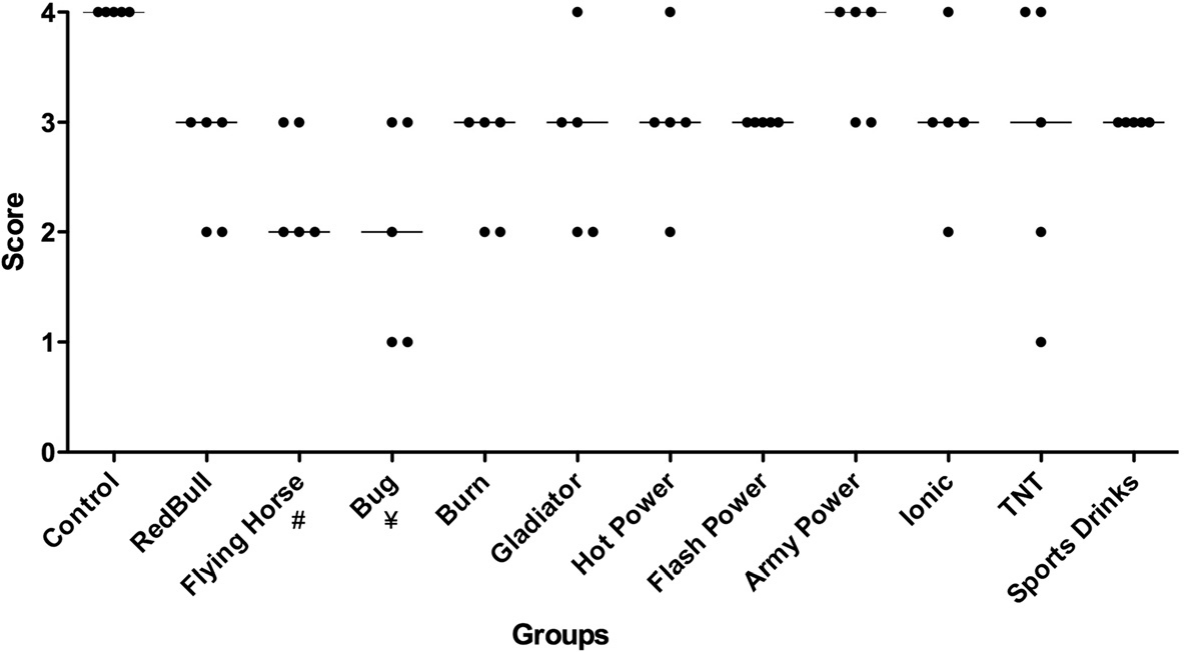
**Frequency distribution of smear layer removal scores in the topical application.** # significant difference in comparison to the control group (*p* < 0.05 – Kruskal-Wallis, Dunn). ¥ significant difference in comparison to the control group (*p* < 0.01 – Kruskal-Wallis, Dunn). The median of all scores is indicated by a line. Individual scores are represented by dots.

Distribution of scores assigned to each group using the friction application are shown in Figure 2. Kruskal-Wallis followed by Dunn’s test showed a significant influence of energy drinks on smear layer removal between the following groups: FlyingHorse™ and control (p < 0.05); Bug™ and control (p <0.001). Army Power™ energy drink was not able to remove the smear layer when applied topically.

**Figure 3:**
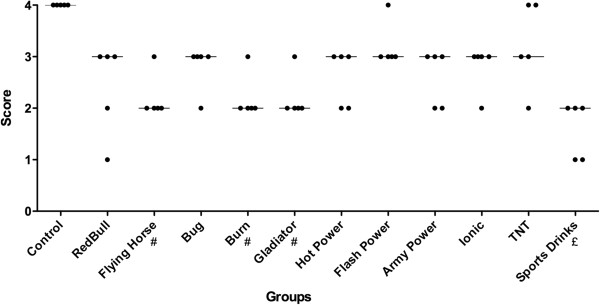
**Frequency distribution of smear layer removal scores in the friction application.** # significant difference in comparison to the control group (*p* < 0.05 – Kruskal-Wallis, Dunn). £ significant difference in comparison to the control group (*p* < 0.001 – Kruskal-Wallis, Dunn). The median of all scores is indicated by a line. Individual scores are represented by dots.

Frequency distribution of scores assigned to each group when it was used as a friction application is showed in Figure 3. Significant differences were observed comparing Burn™ and control (p <0.05), FlyingHorse™ and control (p <0.05), Gladiator™ and control (p <0.05) and between SportsDrinks™ and control (p <0.001). Friction application was able to remove smear layer on the dentine surface for all energy drinks evaluated – scores 2 and 3 (Figure 1).

Comparing the different application forms (topical and friction) for each energy drink tested, a statistically significant difference was found between the two application methods for Army Power™ (p = 0.042) (Table 2). The Army Power™ energy drink has removed the smear layer when friction was applied.

**Table 2:** Smear layer removal data from topical and friction applications

**Group**	**Application method**	**Median (range; lower quartile, upper quartile)**	***#***
Control (Distilled water)	Topical	4.0 (4–4; 4, 4)	ns
	Friction	4.0 (4–4; 4, 4)	
RedBull	Topical	3.0 (2–3; 2, 3)	ns
	Friction	3.0 (1–3; 1.5, 3)	
Flying Horse	Topical	2.0 (2–3; 2, 3)	ns
	Friction	2.0 (2–3; 2, 2.5)	
Bug	Topical	2.0 (1–3; 1, 3)	ns
	Friction	3.0 (2–3; 2.5, 3)	
Burn	Topical	3.0 (2–3; 2, 3)	ns
	Friction	2.0 (2–2; 2, 2.5)	
Gladiator	Topical	3.0 (2–3; 2, 3.5)	ns
	Friction	2.0 (2–3; 2, 2.5)	
Hot Power	Topical	3.0 (2–4; 2.5, 3.5)	ns
	Friction	3.0 (2–3; 2, 3)	
Flash Power	Topical	3.0 (3–3; 3, 3)	ns
	Friction	3.0 (3–4; 3, 3.5)	
Army Power	Topical	4.0 (3–4; 3, 4)	p = 0.04
	Friction	3.0 (2–3; 2, 3)	
Ionic	Topical	3.0 (2–4; 2.5, 3.5)	ns
	Friction	3.0 (2–3; 2.5, 3)	
TNT	Topical	3.0 (1–4; 1.5, 4)	ns
	Friction	3.0 (2–4; 2.5, 4)	
Sports Drink	Topical	3.0 (3–3; 3, 3)	ns
	Friction	2.0 (1–2, 1, 2)	

Mann–Whitney test (*p* = 0.05).

ns = not significant (p > 0.05).

## Discussion

The consumption of energy drinks has increased considerably, mainly amongst young people who are the main consumers of these products [6,11,13] Thus, original research is required to evaluate the erosive potential of energy drinks, since acids in food and beverage products are one of the main factors related to cervical dentine hypersensitivity [14].

The dentine specimens were sectioned tangentially at root dentine to expose the dentinal tubules in the same way when they occur in patients with dental root exposure [15].

After periodontal treatment, an irregular and amorphous layer is created on the root surface called the smear layer. The smear layer can obliterate the dentinal tubules and the dentine fluid movement will be blocked or reduced considerably. The dentine fluid movement is responsible for painful transmission after a stimulus in cervical dentine hypersensitivity [16]. In the present study, smear layer was created by hand instrumentation with periodontal curettes prior to acid drinks exposure.

Eleven different energy drinks were assessed for their erosive potential in dentine and compared with the control group (distilled water). Two different application forms were used: topical and friction. Topical application was used to evaluate the effect of energy drinks on the dentin surface covered with smear layer. Friction application has evaluated the effect of brushing performed immediately after application of the different drinks on the dentinal tubules exposure [14,17]. The dentine specimens were kept immersed in acidic beverages for 5 min, as it is the time necessary for saliva to neutralize and/or remove the acid of the tooth surfaces [18].

Despite the dentine surface showing higher dentinal tubule exposure in specimens immersed in energy drinks, significant statistical differences were only found for Flyinghorse™ and Bug™ energy drinks in comparison with the control group (topical application). In relation to friction application, statistically significant differences (P < 0.05) were observed comparing Burn™ , Flyinghorse™ , Gladiator™ and SportDrinks™ with the control group. According with our results, the null hypothesis tested was partially accepted.

Before immersing the specimens in the energy drinks, the pH were evaluated (Table 1). Although all the energy drinks evaluated have shown a pH below 5.5, which is considered critical for loss from enamel, mineral loss may begin even at higher pH [16]. The lowest pH value was recorded for SportsDrinks™ , however, significant dentinal tubule exposure for this product was found only for the friction application.

The pH is an important factor that may influence dentine erosion. In addition, the pH is easily measured and frequently used to record the acidity of a product [5]. However, pH values give only a measure of initial and dissociated hydrogen ion concentration, therefore, they do not indicate the presence of undissociated acid. Total titratable acidity is the more accurate measure of the total acid content of a drink [19].

There are other features that may influence dentine erosion, such as: buffering capacity of saliva, type of acid, including its chelating properties and sugar content [5,17].

The comparison between application forms has shown higher dentinal tubule exposure for the friction application, although a statistically significant difference was found only for Army Power™ energy drink.

Previous studies have shown that a surface demineralized by acid is vulnerable to toothbrushing, favouring tooth structure removal and dentinal tubule exposure [6,17,20]. Although acid diet associated with or without toothbrushing is able to provoke dentinal tubules exposure, comparison among different acid beverages is not always significant. It may occur due to acid features of each beverage. The type of acid may also influence the erosion potential [5].

Citric acid is also called INS 330 acidulant. This acid is one of the most erosive acids due to its chelating capacity, which is responsible for calcium sequestration from saliva and teeth. Therefore, beverages with low pH and containing citric acid are considered to have the most erosive capacity [21].

The erosive potential showed for energy beverages may be related to presence of citric acid, since all energy drinks evaluated in the present study contain this acid.

In order to reduce the harmful effects on teeth, components have been added to or eliminated from some acid beverages [15]. One of the approaches has been the supplementation with sodium citrate [22].

Studies were conducted to assess the benefits of the acid reduction provided by adding citrate [23,24]. The results show benefits when citrate is used in low concentrations, increasing the pH in the oral cavity, and it can also increase salivary flow, leading to fast elimination of acid after acidogenic challenge [15].

However, sodium citrate has also chelating properties, which could favor the erosive effects. On the other hand, the chelating properties of citrate may be of little importance at the low pH levels of acidic beverages [22]. Future studies should be developed considering the effects of citrate as modifying agents on erosive potential, since it seems to be poorly understood.

Non-reducing sugars of energy drinks may be another factor that explains the difference found for the erosive potential of the energy drinks. Cavalcanti et al. [11] assessed the erosive potential of 9 different energy drinks. Of the drinks analysed (Bad Boy Power Drink™ ; Red Bull™ ; Red Bull Sugar free™ ; Flying Horse™ ; Flying Horse light™ ; Burn™ ; Night Power™ ; Flash Power™ ; 220V Sports Drink™ ), Flying Horse™ showed the highest non-reducing sugar (sucrose – 54.3%) in comparison with the other energy drinks evaluated. This may explain the erosive potential presented by the Flying Horse™ in this study, which showed a significant exposure of dentinal tubules even when it was evaluated topically [11].

Few studies have evaluated the erosive potential and physical-chemical characteristics of energy drinks [11,12], which complicates the comparison of the present study with previous research and also the explanation of the results obtained. Thus, it is important to evaluate the composition of these substances, since the consumption of energy drinks has been increasing over the years.

FlyingHorse™ and Bug™ energy were significantly different from the control group when applied topically, while Burn™ , FlyingHorse™ , Gladiator™ and SportsDrinks™ were significantly different from control when applied by friction (P < 0.05). Comparison between two application forms revealed greater exposure of dentinal tubules when ArmyPower™ was applied by friction. Thus, it is possible to observe from our results the influence of energy drinks as an etiological factor for cervical dentine hypersensitivity.

## Conclusion

Energy drinks can be an important etiological factor for cervical dentine hypersensitivity.

## Competing interests

The authors declare that they have no competing interests.

## Authors’ contribution

CNS carried out the laboratory assays. SCSP conceived the study and helped to draft the manuscript. RC carried out the laboratory assays and performed the statistical analysis. AHB participated in the sequence alignment and drafted the revised manuscript. MCB participated in the design of the study and drafted the manuscript. JECS participated in the design of the study and participated in its design and coordination. All authors read and approved the final manuscript.
